# Genetic risk score predicts risk for overweight and obesity in Finnish preadolescents

**DOI:** 10.1111/cob.12342

**Published:** 2019-10-09

**Authors:** Heli Viljakainen, Emma Dahlström, Rejane Figueiredo, Niina Sandholm, Trine B. Rounge, Elisabete Weiderpass

**Affiliations:** ^1^ Folkhälsan Institute of Genetics Folkhälsan Research Center Helsinki Finland; ^2^ Department of Food and Nutrition University of Helsinki Helsinki Finland; ^3^ Department of Nephrology, Abdominal Center University of Helsinki and Helsinki University Hospital Helsinki Finland; ^4^ Research Program for Clinical and Molecular Metabolism, Faculty of Medicine University of Helsinki Helsinki Finland; ^5^ Faculty of Medicine University of Helsinki Helsinki Finland; ^6^ Department of Research Cancer Registry of Norway, Institute of Population‐based Cancer Research Oslo Norway; ^7^ Department of Medical Epidemiology and Biostatistics Karolinska Institute Stockholm Sweden; ^8^ Department of Community Medicine, Faculty of Health Sciences University of Tromsø, The Arctic University of Norway Tromsø Norway

**Keywords:** BMI, children, genetic risk score, lifestyle factors, waist‐to‐height

## Abstract

Common genetic variants predispose to obesity with varying contribution by age. We incorporated known genetic variants into genetic risk scores (GRSs) and investigated their associations with overweight/obesity and central obesity in preadolescents. Furthermore, we compared GRSs with lifestyle factors, and tested if they predict the change in body size and shape in a 4‐year follow‐up. We utilized 1142 subjects from the Finnish Health in Teens (Fin‐HIT) cohort. Overweight and obesity were defined with age‐ and gender‐specific body mass index (BMI) *z*‐score (BMIz), while central obesity by the waist‐to‐height ratio (WHtR). Background data on parental language, eating habits, leisure‐time physical activity (LTPA) and sleep duration were included. Genotyping was performed with the Metabochip platform. Weighted, standardized GRSs were derived. Of the11‐year‐old children, 25.5% were at least overweight and 90.8% had Finnish speaking background. BMI‐GRS was associated with higher risk for overweight with odds ratio (95% confidence interval) of 1.39 (1.20; 1.60) and obesity 1.41 (1.08; 1.83), but not with central obesity. BMI‐GRS was weakly and inversely associated with the changes in BMIz and WHtR in the 4‐year follow‐up. Waist‐to‐hip ratio‐GRS was not related to any obesity measures at baseline nor in the follow‐up. The effect of BMI‐GRS is similar to that of low LTPA on overweight. An interaction between parental language and BMI‐GRS was noted (*P* = .019): BMI‐GRS associated more strongly with overweight in Swedish than in Finnish speakers. We further identified two suggestive genetic variants near *LOC101926977* and *LOC105369677* associated with BMIz in preadolescents which were replicated in the adult population. In preadolescents, known genetic predisposing factors induce a risk for overweight comparable to low LTPA. However, the GRS was poor in predicting short‐term changes in BMI or WHtR.

**Abbreviations**
BMI: body mass index
BMIz: age‐ and gender‐specific body mass index *z*‐score
GRS: genetic risk score
GWAS: genome‐wide association study
LTPA: leisure‐time physical activity
MAF: minor allele frequency
PC: principal component
QC: quality control
SNP: single nucleotide polymorphism
WHR: waist‐to‐hip ratio
WHtR: waist‐to‐height ratio

## 1 INTRODUCTION

The world is in the midst of an epidemic of obesity, and the number of obese children worldwide has increased to 124 million in 2016.^1^ Data from the prospective Finnish Health in Teens (Fin‐HIT) cohort in 2011 to 2014 showed that about 12.7% of 9‐ to 12‐year‐old Finnish children were overweight, while 2.6% were obese^2^ and similar prevalence was maintained in the follow‐up 3 to 5 years later. Children with overweight have a higher risk for non‐communicable diseases that might also appear at a younger age than in normal‐weight peers.^3^, ^4^ Since early prevention of weight gain is of great importance,^5^ any tools for early identification of persons at risk are valued.

The aetiology of obesity is complex, with many factors involved in its development, for example, lifestyle factors, other environmental factors, genetic susceptibility, and likely their interactions. The era of genome‐wide association studies (GWASs) has increased our knowledge of common genetic variants together explaining about 6% of the variation in adult body mass index (BMI).^6^ Critically considered, the GWASs have not added to our knowledge of predicting BMI beyond maternal BMI,^7^ or other well‐characterized conventional factors, for example, family income, birth weight, high‐sensitive c‐reactive protein.^8^ In turn, several other studies have witnessed that many of the same genetic variants contribute to the variation in child BMI and BMI trajectories from childhood.^9^, ^10^, ^11^ However, their contribution may appear and vary with age and in different stages of life.^11^, ^12^

BMI is a measure of body size reflecting fat accumulation and overall adiposity, while body shape in terms of waist circumference or waist‐to‐hip ratio (WHR) distinguishes fat distribution and serves as markers of central obesity. WHR is independently of BMI associated with an elevated risk for chronic diseases including type 2 diabetes and cardiovascular diseases.^13^ Despite the crudeness of these anthropometric measures, these traits and some of their associated genetic loci have been validated against more sophisticated phenotypes (body fat content or local fat distribution by magnetic resonance imaging).^14^, ^15^ During puberty, body size and fat distribution change dramatically. It is shown that overweight, especially during puberty, modifies the risk of type 2 diabetes in adulthood, while normalization of BMI before puberty may reduce this risk.^16^

In the present study we investigated associations of genetic loci with body size and shape in 1142 Finnish preadolescents and created genetic risk scores (GRSs) for BMI and WHR based on the literature.^17^, ^18^ We hypothesize that the GRSs identify persons with elevated risk for obesity and central obesity. In addition, we compared GRSs with other lifestyle factors and tested if the GRSs predict change in body size and shape in a 4‐year follow‐up study.

## 2 MATERIALS AND METHODS

### 2.1 Study design and population

Material collected in the Fin‐HIT cohort was utilized in the present study. The cohort is described elsewhere in detail.^2^ Shortly, the Fin‐HIT is a prospective cohort consisting of over 11 000 9‐ to 12‐year‐old (born between 1998 and 2006) children and their parents, of which over 90% are participants' biological mothers. Several regions of Finland were represented in the cohort, including Uusimaa, Varsinais‐Suomi, Häme, Pirkanmaa, Keski‐Suomi, Pohjois‐Savo and Pohjois‐Pohjanmaa. Topics covered by child and parent questionnaires are listed on the study website (http://www.finhit.fi/data/). The material collection was conducted at school.

Households with a participating adult and child were invited to the first active follow‐up in 2015 to 2016 when the adolescents were 13‐ to 15‐year‐old. The follow‐up was conducted with an online questionnaire allowing participation at home. All participants gave written informed consent, and the Coordinating research Ethics Committee of the Hospital District of Helsinki and Uusimaa approved the study protocol.

### 2.2 Age, gender and language

Information on gender, date of birth and parental language spoken at home (Finnish, Swedish or other), was obtained from the consent forms or questionnaires and confirmed by linkage with the National Population Information System at the Population Register Centre. Parental language mirrors genetic substructure of the Finnish population. Genetically, the Swedish speakers resembles more closely to European population than Finnish speakers do.^19^ In addition, parental language is a proxy of socioeconomic status with some limitations.^20^, ^21^

### 2.3 Anthropometry/measures of body size and shape

At baseline, trained field workers measured subject's height to the nearest 0.1 cm with a portable stadiometer (Seca model 217) and weight to the nearest 0.01 kg with a portable digital scale (CAS model PB), as explained in detail elsewhere.^2^ BMI was calculated as weight (kg) divided by the square of height (m^2^), and it mirrors body size. BMI was derived into age‐ and gender‐specific BMI *z*‐score (BMIz) according to equations of the International Obesity Task Force (IOTF).^22^ BMIz cut‐offs for underweight, normal‐weight, overweight and obesity were less than −2, between −2 and +1, over +1 and over +2, respectively.

Waist circumference was assessed midway between the hip bone and the ribs to the nearest 0.1 cm with a measuring tape as previously described.^2^ Waist‐to‐height‐ratio (WHtR) was calculated by dividing the waist circumference (cm) by the height (cm), and it illustrates body shape. In children, WHtR mirrors cardiovascular risk factors more accurately than a combination of BMI and waist.^23^ A cut‐off of 0.5 is used for central obesity.^24^, ^25^

In the follow‐up, the child's height, weight and waist were self‐measured and ‐reported by a parent. We have previously reported the validity of the self‐reported anthropometry.^26^

### 2.4 Questionnaire data

#### 2.4.1 Eating habits

Subjects filled in a 16‐item food frequency questionnaire covering the preceding month. The selected food items were adopted from Health Behaviour in School‐Aged Children Study protocol^27^ and these included fruits, fresh or cooked vegetables, sugary soft drinks, dark grain bread, milk or soured milk, fresh juice, water, pizza, hamburger or hot dog, biscuits/cookies, ice cream, chocolate or sweets, salty snacks and sugary juice drinks. Children reported the frequency of consumption for each item on a 7‐point scale ranging from 0 (not consumed) to 6 (consumed several times per day).

Based on food item frequencies, eating habit variable was created utilizing a factor and cluster analyses as explained more in depth elsewhere.^28^ The cluster analysis confirmed three eating habits in the cohort: unhealthy eating habit (loaded with pizza, hamburger or hot dog, baked goods, salty snacks and sugary drinks), fruit and vegetable avoider (avoided unhealthy food items, fruit, berries and vegetables in all forms) and healthy eating habit (loaded with dark grain bread, milk, fruits, berries and vegetables in all forms).

### 2.5 Leisure‐time physical activity

Duration of leisure‐time physical activity (LTPA) was assessed with the question: “How many hours a week do you normally exercise or do sports during your free time?” with 10 response options ranging from “An hour a week or less” to “Around ten hours a week.” We categorized the responses into two categories, less than 7 hours per week or at least 7 hours per week, to mimic the adherence to current guidelines.^29^

### 2.6 Sleep duration

The subjects replied the time asleep on school nights with the question: “When do you usually fall asleep in the evenings on a school night?” with 12 response options. Correspondingly, the waking up on school days was asked: “When do you usually wake up on school days?” with seven response options. Sleep duration on school nights was calculated and categorized into three groups: (a) less than recommended, (b) recommended and (c) more than recommended according to the age‐specific Childhood Sleep Guidelines by the American Academy of Pediatrics^30^: children between 6 and 12 years should sleep daily 9 to 12 hours on a regular basis to promote optimal health. Since we had only three observations in the third category, these subjects were combined into the recommended category.

### 2.7 DNA extraction, genotyping and quality control

All participants of the Fin‐HIT study provided saliva samples using the Oragene DNA Self‐Collection Kit (OG‐500, DNAgenotek). DNA was extracted using an automated protocol with the chemagic DNA Saliva Kit (PerkinElmer, Wellesley, Massachusetts). Altogether 1368 salivary DNA samples were then randomly selected from the Fin‐HIT cohort and genotyped with the Cardio‐Metabochip (Illumina, Inc., San Diego, California) at the Finnish Institute for Molecular Medicine (FIMM) Technology Centre (Helsinki, Finland). Of these 1368 samples, a total of 216 samples were removed after genotyping due to either low sample call rate (<95%) (n = 12), inconsistent gender information (n = 9), duplicate samples/twins (n = 14), relatedness (n = 8) or missing clinical data (n = 173).

After sample quality control (QC), we performed QC on the ~200 000 single nucleotide polymorphisms (SNPs) included on the Metabochip platform. Of these SNPs, we received 195 884 successfully genotyped SNPs from the FIMM Technology Centre. We further excluded 70 697 SNPs that were either mitochondrial or on X/Y chromosome (N = 250), had a low call rate (<95%; N = 4646), deviated from the Hardy‐Weinberg equilibrium (*P* < 10^−6^; N = 514) or had a low minor allele frequency (MAF < 0.01; N = 65 287). Thus, the final analysis set consisted of 125 187 SNPs.

Next, principal component (PC) analysis was performed on the Metabochip SNPs that passed QC to identify ethnicity outliers and to compute PCs to adjust for population stratification. Before calculating PCs, we pruned the SNPs based linkage disequilibrium (LD) using a window size of 1000 kb and a pairwise *r* ^2^ of 0.3 and including only SNPs with MAF >5%. We also removed regions with high levels of LD prior to pruning.^31^, ^32^ The LD pruning resulted in 39 502 variants, which were used to calculate 20 PCs. The PCs were assessed graphically, and ethnicity outliers were defined as ±5SD of the mean of PC1, which explained a majority of the genetic variation in the data. After removal of the ethnicity outliers (n = 10), PCs were recalculated and used in analyses to control for the remaining population stratification.

The number of individuals and SNPs included in the final analysis after QC was thus 1142 and 125 187 with a total genotyping rate of 99.9%. Of these 1142 individuals, 573 (50.2%) were females and 569 males (49.8%). Follow‐up data on BMI and waist circumference was additionally available for 727 individuals (63.7%). QC was performed using plink v 1.09 and R software v 3.5.2.

### 2.8 Creation of genetic risk scores

We selected 32 SNPs for BMI and 32 SNPs for WHR based on the large GWASs performed on these traits at the time of study design in 2010, reflecting also the selection of SNPs to the Metabochip genotyping platform.^17^, ^18^ Of the 32 BMI‐SNPs, 21 were directly available on the Metabochip platform, and a good proxy SNP was available for nine SNPs (*r* ^2^ > 0.80, MAF > 5% or *r* ^2^ = 1.00 if MAF < 5%). Proxy SNPs were derived using LDlink's LDproxy tool, and Finnish population data.^33^ Two BMI SNPs (rs4771122 and rs4836133) were not available and had no good proxies. Thus, the final BMI‐GRS consisted of 30 SNPs (Table S1). Of the 32 WHR SNPs, two were not available on the Metabochip platform (rs9687846 and rs12608504) and had no proxies, and thus, 30 SNPs were included in the WHR‐GRS.

We created weighted GRSs using the score function in Plink v1.09, which calculates an average score per non‐missing SNP. This score is calculated by multiplying the weight of each SNP with the number of risk alleles for that SNP. As weights, we used the effect sizes from the original publications for BMI (Speliotes et al,^17^ Table S1) and WHR (Heid et al,^18^ Table S2). Finally, we standardized the GRS using the mean and SD in the sample. Since there were more recent and larger GWASs available^6^, ^7^, ^34^ than the one of Speliotes et al,^17^ we reconstructed BMI‐GRSs based on the different sources and compared their performance in our sample (Table S3). As the BMI‐GRS based on Speliotes et al explained the highest amount of variance in the BMIz with the smallest *P*‐value, we used it solely in the further analyses.

### 2.9 Statistical analyses

#### 2.9.1 Genetic association analyses

We performed metabochip‐wide association studies for BMIz and standardized residuals of WHtR after adjusting for age and gender. The association was tested individually for each SNP with an additive model (coded as the number minor alleles [0, 1, 2]) for BMI and WHtR against the outcome variable using linear regression. The first two PCs mirroring the genetic substructure of the population were included as covariates in the model. The genetic associations were tested in Plink v.1.09. Power analyses were conducted in R using package “pwr.” To determine the *P*‐value threshold for metabochip‐wide significance, SNPs were LD pruned (window size: 1000 kb, pairwise *r* ^2^ of 0.8, MAF > 1%) in order to calculate the number of independent SNPs (n = 68 183). The *P*‐value threshold for a significant finding was thus 7.0 × 10^−7^, which is a 5% Bonferroni‐corrected threshold for 68 183 independent SNPs. We defined a suggestive hit as *P* < 1.0 × 10^−4^.

#### 2.9.2 Other statistical analyses

The normal distribution of variables was visually inspected, and transformations applied when needed. Comparison of baseline characteristics between two groups was performed with independent samples *t* test in case of a continuous variable, and with chi‐square test for categorical variables.

Logistic regression was used to study the association of GRSs with overweight (BMIz > +1), obesity (BMIz > +2) and central obesity (WHtR > 0.5) in two models: first GRS alone adjusted for PC1 and PC2, and a second multivariate model including GRS with all covariates, and adjusted for age, PC1 and PC2. Covariates in the analysis were gender, parental language, LTPA, sleep duration, and eating habits. Female gender, Finnish language, LTPA >7 hours per week, recommended sleep duration and healthy eating habit were considered as reference categories in the analysis. The associations were reported as odds ratio (OR) with 95% confidence interval (CI).

We had missing values in several covariates: parental language (n = 33), LTPA (n = 11), eating habit (n = 109) and sleep (n = 59). The missing values were replaced in logistic regression analysis using the multiple imputation procedure in SPSS, in order to maintain the full sample size. The imputation method was “fully conditional specification,” which suits for arbitrary missing data. Multiple variables, for example, all covariates, GRSs and outcome measures in original scale, were included in the imputation process.

Associations of covariates (gender, parental language, LTPA, sleep duration, eating habit) with the two GRSs were tested with *t* test or analysis of variance.

Association of GRSs with changes in BMIz and WHtR from baseline to 4‐year follow‐up were investigated with linear regression in a subgroup of 727 subjects. Model 1 was adjusted for gender, age, sleep duration, eating habit, LTPA, parental language, PC1 and PC2. Model 2 included an additional adjustment for baseline BMIz or WHtR.

Interactions between covariates and GRSs were tested with log‐likelihood ratio comparing models with and without interaction terms.

All statistical analyses were conducted using the IBM SPSS program for Windows, version 22 (IBM, Chicago, Illinois). The statistical significance level was set at 5%.

## 3 RESULTS

### 3.1 Baseline characteristics

Of the total of 1142 subjects, 8 (0.7%) were categorized as underweight, 843 (73.8%) normal weight, 230 (20.1%) overweight and 61 (5.3%) obese based on IOTF age‐ and gender‐specific BMIz. A combined prevalence of overweight and obesity was more common in boys than in girls (29.7% vs 21.3%, *P* = 0.001), while the prevalence did not differ by parental language: 25.2%, 30.8% and 20.8% with Finnish, Swedish and other language family background, respectively (*P* = .732). The subjects were recategorized into two groups combining under‐ and normal‐weight into “UW/NW” (BMIz ≤ +1) and overweight and obese into “OW/OB” (BMIz > +1) (Table 1). As expected, weight, waist, BMI, WHtR and parental BMI were higher in OW/OB than in UW/NW group, while no differences were observed in height or age. Male subjects and lower physical activity level were more commonly seen in OW/OB than UW/NW group.

**Table 1:** Background descriptive factors by groups of under‐/normal‐weight (UW/NW) and overweight/obese (OW/OB) with mean (SD), if not indicated otherwise

	UN/NW (BMIz ≤ +1), n = 851		OW/OB (BMIz > +1), n = 291		
	Mean	SD	Mean	SD	*P*‐value^a^
Age, y	11.3	(0.2)	11.3	(0.2)	.335
Weight, kg	37.1	(5.0)	50.7	(7.8)	<.001
Height, cm	147.9	(6.6)	151.2	(6.6)	<.001
BMI, kg/m^2^	16.9	(1.5)	22.1	(2.4)	<.001
BMI *z*‐score	−0.1	(0.7)	1.6	(0.5)	<.001
Waist, cm	63.4	(4.8)	75.8	(8.2)	<.001
Waist‐to‐height ratio	0.43	(0.03)	0.50	(0.05)	<.001
Parental BMI, kg/m^2^ (n = 949)	24.4	(4.3)	26.4	(4.4)	<.001
Weighted GRS of WHR, *z*‐scored	0.01	(1.0)	−0.04	(1.0)	.393
Weighted GRS BMI, *z*‐scored	−0.08	(1.0)	0.23	(1.0)	<.001
	n	%	n	%	
Gender					.001^b^
Girls	451	53.0	122	41.9	
Boys	400	47.0	169	58.1	
Parental language (n = 1109)					.483^b^
Finnish	753	91.2	254	89.8	
Swedish	54	6.5	24	8.5	
Other	19	2.3	5	1.8	
Eating habit (n = 1033)					.387^b^
Unhealthy	101	13.1	38	14.4	
Vegetable and fruit avoider	330	42.9	122	46.4	
Healthy	339	44.0	103	39.2	
Leisure time physical activity (n = 1131)					.019^b^
<7 h per week	368	43.6	148	51.6	
7 h per week or more	476	56.4	139	48.4	
Sleep duration during week (n = 1083)					.474^b^
Less than recommended	26	3.2	13	4.7	
Recommended	781	96.5	260	94.9	
More than recommended	2	0.2	1	0.4	

Abbreviations: BMI, body mass index; WHR, waist‐to‐hip ratio; GRS, genetic risk score.

a *t* test.

b Chi‐square test.

Follow‐up measurements were available from 727 subjects, and their baseline characteristics were similar to those of 1142 subjects (Table S3). During the follow‐up, weight increased similarly in the two groups, but a higher increment in height (22.1 vs 20.4 cm, *P* = .005) and waist circumference (8.5 vs 5.6 cm, *P* < .001) were observed in UW/NW than in OW/OB group. BMIz increased among those in UW/NW group, while decreased in OW/OB group. In both groups, the WHtR decreased, but more so in OW/OB group.

### 3.2 Genetic risk scores

BMI‐GRS was higher in the OW/OB than in the UW/NW group (*P* < .001), while no group difference was observed for WHR‐GRS (Table 1). With our sample size we had 81% power to detect an explained variance (*R* ^2^) of 1.0% and 95% power to detect *R* ^2^ of 1.5%. The BMI‐GRS in our sample was associated with BMIz (*P* = 6.2 × 10^−11^) and it explained 3.7% of the variance in BMIz (after adjusting for PC1and PC2). Correspondingly, BMI‐GRS was associated with BMIz at follow‐up (*P* = .018) explaining 0.4% of the variance. The WHR‐GRS was not associated with WHtR at baseline or follow‐up (*P* = .40, *R* ^2^ = 0.06% and *P* = .56, *R* ^2^ = 0.02%).

### 3.3 Associations of GRSs with overweight, obesity and central obesity at baseline

Associations of GRSs with overweight (BMIz > +1), obesity (BMIz > +2) and central obesity (WHtR ≥ 0.5) were tested with logistic regression in two models (Tables 2 and 3). An SD‐increase in weighted BMI‐GRS increased the risk for overweight with an OR of 1.39 (95% CI: 1.21; 1.60) and obesity with 1.41 (95% CI: 1.08; 1.83). WHR‐GRS was not associated with any of the obesity estimates.

**Table 2:** Association of BMI‐GRS with overweight and obesity combined, obesity alone, and central obesity in univariate and multivariate models^a^ with OR (95% CI)

	Overweight + obesity^b^								Obesity^c^								Central obesity^d^							
	Univariate				Multivariate				Univariate				Multivariate				Univariate				Multivariate			
	OR	95% CI		*P*‐value	OR	95% CI		*P*‐value	OR	95% CI		*P*‐value	OR	95% CI		*P*‐value	OR	95% CI		*P*‐value	OR	95% CI		*P*‐value
BMI‐GRS, *z*‐score	1.38	1.20	1.58	*<.001*	1.39	1.21	1.60	*<.001*	1.40	1.08	1.81	*.012*	1.41	1.08	1.83	.011	1.18	1.00	1.40	.056	1.18	0.99	1.40	.064
Sex																								
Female					1.00								1.00								1.00			
Male					1.63	1.23	2.15	*.001*					2.20	1.26	3.86	*.006*					1.81	1.27	2.58	*.001*
Parental language																								
Finnish					1.00								1.00								1.00			
Swedish					1.10	0.63	1.93	.731					0.89	0.29	2.80	.846					0.69	0.33	1.47	.337
Other					0.41	0.15	1.14	.087					0.54	0.07	4.50	.571					0.72	0.23	2.30	.582
LTPA																								
≥7 h/wk					1.00								1.00								1.00			
<7 h/wk					1.42	1.07	1.88	*.015*					1.47	0.86	2.52	*.161*					1.82	1.28	2.60	*<.001*
Sleep during week																								
Recommended					1.00								1.00								1.00			
Less than recommended					1.58	0.81	3.08	.179					1.31	0.38	4.55	.674					1.71	0.78	3.75	.177
Eating habits																								
Healthy eating					1.00								1.00								1.00			
Fruit and vegetable avoiders					0.99	0.64	1.53	.953					1.49	0.64	3.48	.357					1.53	0.90	2.60	.117
Unhealthy eating					1.15	0.85	1.55	.36					1.79	0.98	3.27	.06					1.48	1.01	2.18	*.046*

Abbreviations: BMI, body mass index; CI, confidence interval; GRS, genetic risk score; LTPA, leisure‐time physical activity; OR, odds ratio; PC, principal component.

a All models are adjusted for age, PC1 and PC2.

b Defined with BMI *z*‐score greater than +1, n = 291.

c Defined with BMI *z*‐score greater than +2, n = 61.

d Defined with waist‐to‐height ratio ≥0.5, n = 158.

**Table 3:** Association of WHR‐GRS with overweight and obesity combined, obesity alone, and central obesity in univariate and multivariate models^a^ with OR (95% CI)

	Overweight + obesity^b^								Obesity^c^								Central obesity^d^							
	Univariate				Multivariate				Univariate				Multivariate				Univariate				Multivariate			
	OR	95% CI		*P*‐value	OR	95% CI		*P*‐value	OR	95% CI		*P*‐value	OR	95% CI		*P*‐value	OR	95% CI		*P*‐value	OR	95% CI		*P*‐value
WHR‐GRS, z‐score	0.95	0.83	1.08	.417	0.94	0.82	1.07	.332	1.02	0.79	1.32	*0.88*	1.00	0.77	1.30	.992	1.04	0.88	1.23	0.683	1.02	0.86	1.21	.819
Sex																								
Female					1.00								1.00								1.00			
Male					1.62	1.23	2.14	*.001*					2.18	1.24	3.82	*.006*					1.82	1.28	2.59	*.001*
Parental language																								
Finnish					1.00								1.00								1.00			
Swedish					1.06	0.61	1.84	.839					0.87	0.28	2.72	.815					0.77	0.39	1.55	.47
Other					0.41	0.15	1.16	.093					0.55	0.07	4.59	.577					0.76	0.26	2.24	.622
LTPA																								
≥7 h/wk					1.00																1.00			
<7 h/wk					1.45	1.10	1.91	*.009*					1.51	0.89	2.59	.129					1.90	1.33	2.70	*<.001*
Sleep during week																								
Recommended					1.00																1.00			
Less than recommended					1.51	0.79	2.91	.214					1.27	0.37	4.36	.703					1.63	0.75	3.52	.214
Eating habits																								
Healthy eating					1.00								1.00								1.00			
Fruit and vegetable avoiders					0.99	0.64	1.526	.957					1.46	0.63	3.42	.38					1.51	0.89	2.56	.128
Unhealthy eating					1.15	0.852	1.539	.368					1.77	0.97	3.23	.064					1.48	1.00	2.17	*.048*

Abbreviations: CI, confidence interval; GRS, genetic risk score; LTPA, leisure‐time physical activity; OR, odds ratio; PC, principal component; WHR, waist‐to‐height ratio.

a All models are adjusted for age, PC1 and PC2.

b Defined with BMI *z*‐score greater than +1, n = 291.

c Defined with BMI *z*‐score greater than +2, n = 61.

d Defined with waist‐to‐height ratio ≥0.5, n = 158.

### 3.4 Associations of GRSs with changes in BMI *z*‐score and weight‐to‐height ratio

The associations of GRSs with changes in BMIz and WHtR ratio were examined with linear regression in a sub‐sample of 727 subjects with follow‐up data available. For higher BMI‐GRS, we observed a smaller increase in BMIz (*P* = .012) or in WHtR (*P* = .042) in the 4‐year follow‐up. However, the estimated effect sizes were relatively small, and the adjusted models were poor (*R* ^2^ < 0.04). An additional adjustment for baseline BMIz or WHtR overruled the effects of BMI‐GRS. The WHR‐GRS was not associated with changes in BMIz or WHtR (Table S4).

### 3.5 Associations of GRSs with covariates

GRSs did not differ by gender (*P* = .99 and *P* = .61, for BMI‐GRS and WHR‐GRS, respectively), parental language (*P* = .46 and *P* = .17), LTPA (*P* = .22 and *P* = .72), eating habits (*P* = .56 and *P* = .88) or sleep duration (*P* = .23 and *P* = .46).

### 3.6 Interaction with parental language

**Figure 1:**
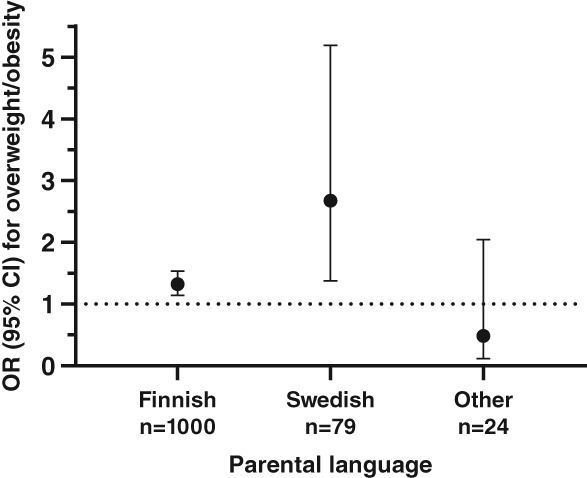
Association of BMI‐GRS with overweight differs by parental language; the strongest association was observed in those with Swedish speaking background. Logistic regression model is adjusted for gender, age, sleep duration during week, eating habit, leisure‐time physical activity, principal components 1 and 2

Interactions were tested between GRSs and sex, parental language, LTPA, sleep, and eating habits in all models regarding overweight, obesity and central obesity. An interaction appeared between parental language and BMI‐GRS concerning overweight/obesity (*P* = .019) (Figure 1).

### 3.7 Top SNPs related to BMI *z*‐score and waist‐to‐height ratio in preadolescents

In metabochip‐wide association study for BMIz and WHtR, no locus reached metabochip‐wide significance (*P* < 7.0 × 10^−7^). In total, 12 SNPs reached *P*‐value of <1 × 10^−4^ for BMIz (Table 4, Figure S1), and 16 SNPs for WHtR (Table 5, Figure S2) and these were considered as suggestive hits. Next, we looked up our suggestive SNPs for BMI in previous large‐scale GWASs^6^, ^7^, ^17^, ^18^, ^34^ (Table 4). Of these, rs12680842 near *LOC101926977* and rs10840674 near *LOC105369677* were associated with BMI also in the most recent GWAS with ~700 000 adults^6^: the former reaching genome‐wide significance (Table 4). In addition, nominal associations (*P* < .05) in GWAS on BMI and WHR from the adult population was obtained for rs9882235 (*ADAMTS9‐AS2*; *P* = .0043^18^) and rs3745010 (*SLC14A2*; *P* = 0.004^6^). None of the variants were associated with BMI in young children in the EGG Consortium^34^ (Table 4).

**Table 4:** Most significant metabochip‐wide loci associated with BMI *z*‐score (*P* < 10^−4^), N = 1142

CHR	BP	SNP	Nearest gene	EA	Effect	EAF	*P*‐value	Metabochip info	*P*_BMI _Speliotes_ a	*P*_WHRadjBMI_Heid_ b	*P*_childhoodBMI_Felix_ c	*P*_BMI_Yengo_ d	*P*_BMI_Locke_ e
1	228 375 007	rs12127595	*GALNT2*	A	−0.18	0.26	9.7 × 10^−5^	HDL cholesterol (F)	NA	NA	NA	NA	NA
3	65 003 792	rs9882235	*ADAMTS9‐AS2*	A	0.18	0.38	1.7 × 10^−5^	Waist‐to‐hip ratio (R)	.66	.0043	.60	.22	.26
3	124 669 753	rs76458540	*ADCY5*	G	0.56	0.02	5.9 × 10^−5^	Fasting glucose (F)	NA	NA	NA	NA	NA
4	175 297 195	rs6827803	*LINC02268*	A	0.17	0.49	3.7 × 10^−5^	Fasting insulin (R)	.14	.46	.32	NA	.33
4	186 637 186	rs6833729	*CCDC110*	A	−0.37	0.06	6.7 × 10^−5^	HDL cholesterol (R)	.85	.34	.25	.51	.81
4	186 706 487	rs1158465	*PDLIM3*	A	−0.17	0.49	5.4 × 10^−5^	Waist circumference (R)	.54	.14	.10	.19	.57
7	79 691 383	rs10247757	*GNAI1*	A	−0.23	0.16	3.7 × 10^−5^	HDL cholesterol (R)	.79	.89	.43	.33	.98
8	95 651 782	rs12680842	*LOC101926977*	G	−0.18	0.39	2.5 × 10^−5^	BMI (R)	.0022	.50	.49	4.4 × 10^−14^	1.7 × 10^−5^
9	77 986 751	rs10869722	*PCSK5*	G	0.16	0.45	8.5 × 10^−5^	Wildcard SNP	.38	.16	.85	NA	.091
10	125 477 905	rs12358957	*CPXM2*	A	−0.18	0.46	3.8 × 10^−5^	Fasting glucose (R)	.79	.92	.58	.43	.21
12	17 251 521	rs10840674	*LOC105369677*	G	0.18	0.33	3.1 × 10^−5^	BMI (R)	.0076	.06	.14	4.5 × 10^−7^	.020
18	41 376 472	rs3745010	*SLC14A2*	A	−0.21	0.18	9.1 × 10^−5^	Diastolic BP (R)	.015	.45	.18	.004	.14

*Note*: Tests were adjusted for principal components 1 and 2. Metabochip info field contains the selection criteria for the metabochip platform.

Abbreviations: F, SNP included for fine‐mapping; NA, not available; R, SNP included for replication.

a Speliotes et al,^17^ N = 123 865.

b Heid et al,^18^ N = 77 167.

c Felix et al,^34^ N = 35 688.

d Yengo et al,^6^ N ~700 000.

e Locke et al,^7^ N ~322000.

**Table 5:** Most significant metabochip‐wide loci associated with waist‐to‐height ratio (*P* < 10^−4^), N = 1142

CHR	BP	SNP	Nearest gene	EA	Effect	EAF	*P*‐value	Metabochip info	*P*_Speliotes_2010_ a	*P*_Heid_2011_ b	*P*_Shungin_2015_ c	*P*_Pulit_2019_ d
1	55 301 217	rs12067569	*PCSK9*	A	0.5	0.03	7.1 × 10^−5^	LDL cholesterol (R)	.51	.98	.76	.09
2	33 232 767	rs6715793	*LTBP1*	G	0.18	0.42	1.8 × 10^−5^	Waist circumference (R)	.16	.0031	NA	.00011
2	33 258 655	rs6751657	*LTBP1*	A	0.17	0.41	4.5 × 10^−5^	Height (R)	.42	.044	.0081	.00027
2	33 285 705	rs4670307	*LTBP1*	A	0.18	0.38	1.1 × 10^−5^	LDL cholesterol (R)	.96	.029	.022	.03
5	116 912 917	rs265893	*LINC00992*	G	−0.17	0.37	8.9 × 10^−5^	Systolic BP (R)	.75	.76	.74	.35
6	13 379 909	rs115483154	*PHACTR1*	A	0.59	0.02	7.8 × 10^−5^	MI‐CAD (F)	NA	NA	NA	.2
7	31 958 698	rs2008835	*PDE1C*	C	0.45	0.04	5.2 × 10^−5^	BMI (R)	.0052	.86	.87	.31
7	70 711 575	rs6979843	*GALNT17*	G	−0.17	0.32	8.8 × 10^−5^	Waist circumference (R)	.76	.0065	.13	.48
7	72 658 273	rs3812316	*MLXIPL*	C	0.25	0.13	8.0 × 10^−5^	Triglycerides (R)	NA	NA	.043	4.6 × 10^−12^
7	153 653 744	rs7808911	*DPP6*	A	−0.3	0.09	1.1 × 10^−5^	CAD (R)	.058	.8	.74	.22
7	153 663 311	rs11771921	*DPP6*	C	−0.28	0.11	1.6 × 10^−5^	BMI (R)	.011	.28	.37	.2
8	12 742 545	rs11774014	*LOC340357*	C	0.29	0.1	6.0 × 10^−5^	CAD (R)	.37	.63	.087	.28
10	125 477 905	rs12358957	*LOC105378532*	A	−0.18	0.46	5.7 × 10^−5^	Fasting glucose (R)	.21	.92	.8	.0038
14	50 062 686	rs7160618	*MAP4K5*	A	0.46	0.04	1.1 × 10^−5^	2‐hour glucose (R)	.22	.033	.034	.12
17	35 005 531	chr17:35005531	*NEUROD2*	C	0.56	0.03	3.6 × 10^−5^	HDL cholesterol (F)	NA	NA	NA	NA
20	45 037 243	rs1150442	*EYA2*	A	0.18	0.03	6.6 × 10^−5^	Triglycerides (R)	.04	.63	.014	1.4 × 10^−6^

*Note*: Tests were adjusted for principal components (PC) 1 and 2. Metabochip info field contains the selection criteria for the metabochip platform.

Abbreviations: F, SNP included for fine‐mapping; MI‐CAD, myocardial infarction, Coronary artery disease; NA, not available; R, SNP included for replication.

a Speliotes et al,^17^ N = 123 865.

b Heid et al,^18^ N = 77 167.

c Shungin et al,^35^ N = 224 459.

d Pulit et al,^36^ N = 484 563.

**Figure 2:**
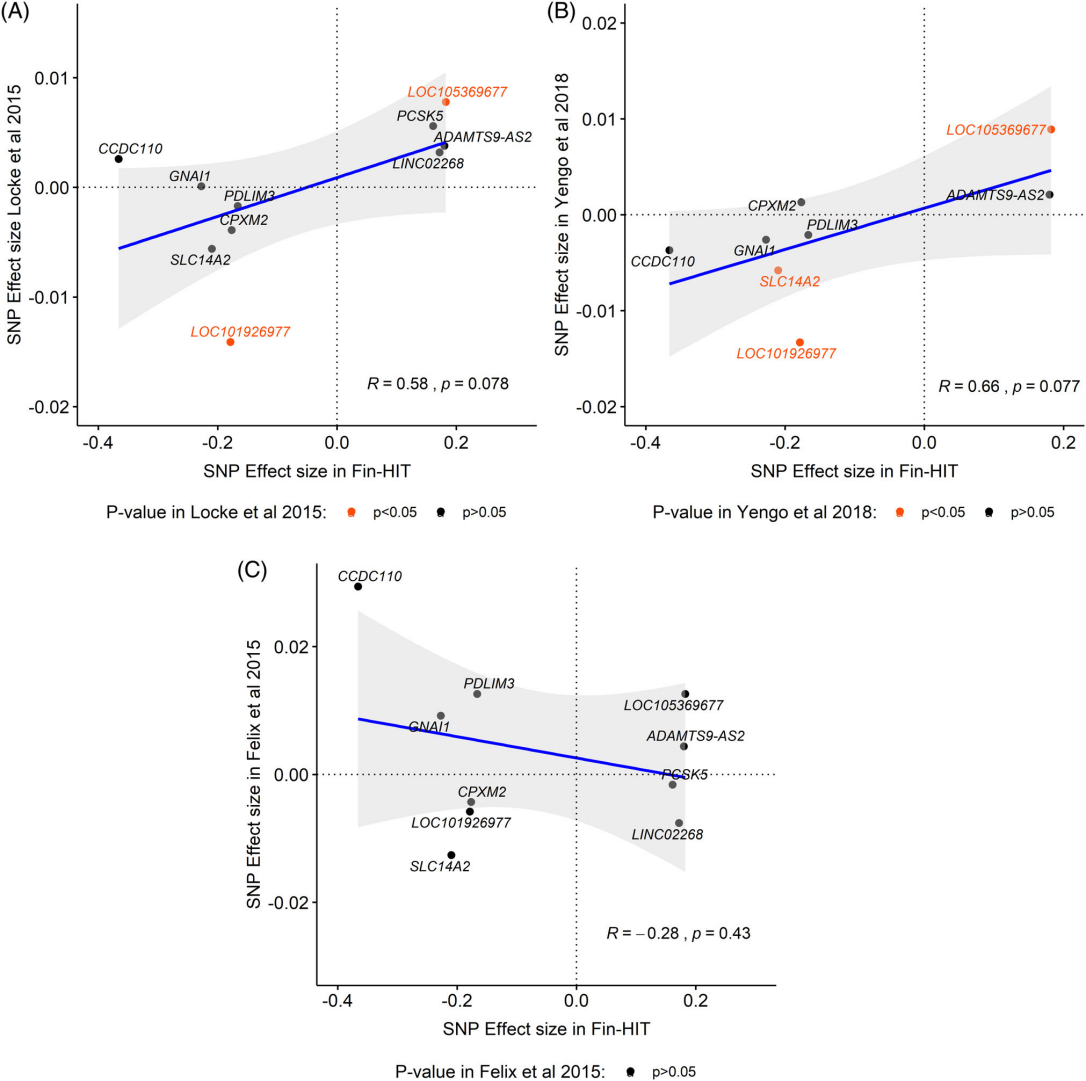
Effect size comparison of the Fin‐HIT suggestive SNPs for BMIz in adult population and in young children

We further tested the correlation between the effect size estimates of the suggestive SNPs found for BMIz in our study and in previous GWASs on BMI.^6^, ^7^, ^34^ Borderline significant (*r* = 0.6, *P* = .08) positive correlations were observed with SNP effects in the two adult population studies, while the effect sizes did not correlate with estimates for BMI in young children from the EGG Consortium (*P* = .43; Figure 2).

Of the 16 suggestive SNPs associated with WHtR, variants in or near *LTBP1*, *PDE1C*, *GALNT17*, *DPP6*, *MAP4K5* and *EYA2* were nominally associated with BMI or WHR in adult population^17^, ^18^, ^35^, ^36^ (Table 5). Of these, highly significant replications were marked for rs3812316, near gene *MLXIPL* (*P* = 4.6 × 10^−12^) and rs1150442 near gene *EYA2* (*P* = 1.4 × 10^−6^) in the recent study of Pulit et al.^36^

## 4 DISCUSSION

Our study shows that a GRS based on well‐defined BMI‐related SNPs (BMI‐GRS) was associated with overweight and obesity in 1142 Finnish preadolescents. The classification of overweight and obesity was based on age‐ and sex‐specific BMIz according to IOTF guidelines.^22^ On the other hand, WHR‐GRS was vague and not related to central obesity defined by the WHtR in the same group. When comparing the effect of BMI‐GRS with lifestyle factors (LTPA, sleep duration and eating habits) in preadolescents, the effect of BMI‐GRS on overweight/obesity was comparable with low LTPA level.

Recent GWAS studies have resulted in the identification of numerous common genetic variants, SNPs, which have been linked with BMI in large datasets. Recently, over 900 SNPs have been robustly associated with adult BMI.^6^ However, with the increasing number of BMI‐related SNPs, the most recent ones demonstrate smaller effect sizes than the earlier ones.^37^ In the present study we utilized Metabochip, a custom genotyping chip for fine‐mapping and replication, which includes altogether 200 000 SNPs from loci that were associated, or were promising for several complex metabolic traits for example, type 2 diabetes, cardiovascular diseases, and their key risk factors: BMI, blood glucose, insulin land lipid concentrations and blood pressure back in 2012.^38^ We created BMI‐GRSs based on data of Speliotes et al,^17^ and 30 of these 32 SNPs were incorporated in Metabochip. The BMI‐GRS was associated with risk of overweight and obesity, and explained as much as 3.7% of BMIz at the baseline, but was not informative regarding the risk of central obesity. Previously, GRS applied to paediatric data has been reported to predict adulthood obesity efficiently 20 to 30 years later.^8^ However, different genetic factors may affect the short‐term changes in BMI, especially during rapid growth period.^11^

Besides body size, we were interested in body shape in terms of central obesity, which has been linked to cardiovascular diseases and diabetes more strongly than BMI.^13^ While WHR has been used as a marker of central obesity in adult GWA studies, we utilized WHtR as a tool for determining central obesity in children in the present study.^23^ WHtR and waist circumference are considered comparable predictors of diabetes and cardiovascular disease, both being stronger than, and independent of, BMI.^23^ For the WHR‐GRS altogether 30 of the total 32 SNPs characterized by Heid et al^18^ were located in Metabochip. However, this GRS was not associated with any obesity outcomes in the baseline or follow‐up. One explanation for our null finding may be that genes relevant to body shape are activated during later stages of life. However, obesity and especially body shape are complex traits, and other factors beyond genetics are also involved.

Fin‐HIT cohort is a well‐defined epidemiological cohort with multiple self‐reported lifestyle factors. Our analysis was adjusted for gender, parental language, eating habits, sleep duration during the week, and LTPA. In present study being a male bears a higher risk for excess weight than the genetic susceptibility. Male gender presented with a higher risk for overweight, obesity and central obesity compared with females, which is in accordance with previous results in this age group^39^, ^40^: the prevalence of overweight and obesity are reported to be higher in boys than in girls in Finland. A higher prevalence of overweight and obesity in boys than in girls at this age seems a common observation,^1^ although not consistent across the globe. Differences in the prevalence of excess weight appear after childhood, suggesting that growth pattern and hormonal changes play a role, which is also supported by the fact that similar or even higher prevalence is reported in girls at younger age groups.^41^ Sex differences may also be related to life‐style factors and beliefs in children and their parents.^42^ Previously, we have shown that unhealthy eating habit was more common in boys than in girls,^28^ while typically boys are more physically active at this age^43^, ^44^ providing no simple explanation for the phenomenon.

Exercising less than 7 hours per week led to a higher risk for overweight and central obesity in our children, which is in line with current physical activity guidelines.^29^ Interestingly, low LTPA and BMI‐GRS have comparable effects on the risk of overweight/obesity. However, for the risk of central obesity, low exercise level had even greater effect than BMI‐GRS. In these 1142 children, eating habits and sleep duration were not related to excess weight. Our study was underpowered to dig into interactions between GRS and lifestyle factors, which are fine‐tuning the effect of genetic factors between individuals. Previously, interactions of alcohol consumption, physical activity, mental health, and sleeping patterns with GRS affecting BMI have been identified.^45^ Of these, physical activity has shown consistent results in various populations (European ancestry and Latin/Hispanic), suggesting two things: (a) that individuals with lower physical activity and more sedentary behaviour present a higher genetic susceptibility to obesity,^46^, ^47^, ^48^ and (b) those with the highest genetic susceptibility to obesity would benefit from increasing physical activity and decreasing sedentary behaviour.

We observed an interaction between GRSs and parental language. In the present study, parental language mirrors socioeconomic status as previously suggested.^21^, ^49^ Since participants of Fin‐HIT are recruited mostly outside the capital region (Helsinki area), differences in socioeconomic position (eg. educational level, household income and poverty) are likely to favour the Finnish speakers. The BMI‐GRS showed the highest risk for excess weight in Swedish speakers, followed by Finnish speakers, while no association was observed in those with other language backgrounds. In UK Biobank material, socioeconomic status was suggested to mimic the obesogenic environment,^45^ which emphasized the effect of genetic risk factors, and corresponding is observed here. Moreover, the association of GRS appears stronger in the more recent US birth cohort compared with a previous birth cohort from the same country, highlighting the role of obesogenic environment as well.^50^ In the present study, 30.8% of children with Swedish speaking background were at least overweight, while the corresponding number for children with Finnish speaking background was 25.2%. In addition, a higher portion of children with Swedish background had low LTPA level than children with Finnish speaking background (62% vs 44%). Evidently, parental language captures several relevant risk factors for overweight in the present study.

We were able to incorporate follow‐up data of 727 subjects with an average follow‐up time of 4 years. Thus, if the subjects were considered preadolescent at the age of 11 years, they become adolescent at the age of 15. Several other studies in children have demonstrated that comparable BMI‐GRSs associate and predict adult weight reliably.^8^, ^10^ However, in our study, the BMI‐GRS was poor in predicting short‐term changes in BMIz or WHtR. Surprisingly, higher BMI‐GRS was associated with smaller changes in BMI and WHtR, but not after further adjustment for baseline measures. This is further supported by our data pointing that during the 4‐year follow‐up the BMI was to some extent normalized in overweight/obese group, while this continued to increase in under‐/normal‐weight group. This implies that growth speed varies by age. It seems the overweight/obese had grown at earlier state compared with under‐/normal‐weight group, as suggested before.^11^, ^51^

Metabochip was utilized here to discover potential novel genetic variants for BMIz in preadolescents. Even though our metabochip‐wide association study was underpowered, we identified 12 + 16 suggestive SNPs associated with *P* < 10^−4^ with BMIz and WHtR, respectively. Two BMI‐related SNPs (rs12680842 and rs10840674) were robustly replicated in adult population,^6^ while several other SNPs reached nominal replication in the adult population as well. Of note, none of the suggestive SNPs were associated with BMI in the EGG consortium GWAS in children aged between 2 and 11 years.^34^ Furthermore, the effect size estimates of the suggestive SNPs for BMIz correlated with the corresponding ones in the adult population,^6^, ^7^ but not with effect size estimates in young children,^34^ which suggest that genetic factors related to body size in preadolescents are more similar to adults than to younger children.

The study had several limitations. The sample size limited the power of metabochip‐wide association analysis, and only suggestive associations were identified. The Metabochip contained over 65 000 SNPs with MAF < 1% in this Finnish study, and those were excluded as rare SNPs were not of interest here. The Metabochip platform is not a fully genome‐wide genotyping platform as it contains markers only in some regions of the genome, for example, regions selected for fine‐mapping and replication based on GWASs on metabolic traits performed before 2012. Thus, it provides less data required for constructing the LD patterns, which is important for genotype imputation.^52^ Regarding the phenotype, the secondary trait in our study was WHtR, which differs from WHR that has been used as a marker of central adiposity in adults,^18^ and a discrepancy between the traits may cause some inaccuracy. An additional limitation was that we had follow‐up data only on 63% of the participants. At follow‐up, the anthropometric measures were self‐assessed, which might cause some bias. Previously, we have validated the self‐reported measures^26^: home‐measured mean height, weight and waist circumference were slightly higher, but derived BMI lower than measured by the fieldworker. However, the differences were so small that they had no impact on weight status. The sampling for Metabochip array was random, but the participants in the present study showed a somewhat higher prevalence of overweight/obesity than seen in the entire cohort, but still comparable with the Finnish population at this age.^39^, ^40^

We have demonstrated that BMI‐GRS is a significant risk factor for overweight and obesity in preadolescents, but is poor in predicting short‐term changes in body size and shape during puberty. The associations of BMI‐GRS and low physical activity with overweight/obesity were similar, but somewhat weaker than being a male. An interaction with socioeconomic status was observed, suggesting that predisposing genetic factors appear more strongly in Swedish than in Finnish speaking children, which still warrants further investigations.

## CONFLICT OF INTEREST

No conflict of interest was declared.

## Supporting information


**Table S1.** SNPs included in the BMI‐GRS used in the study.
**Table S2.** SNPs included in the WHR‐GRS used in the study.
**Table S3.** BMI‐GRS associations with BMI *z*‐score.
**Table S4.** Background characteristics and change in anthropometric measures in 727 subjects by groups of under‐/normal‐weight (UN/NW) and overweight/obese (OW/OB) with mean (SD), if not indicated otherwise.
**Table S5.** Associations of GRSs with change in BMI *z*‐score and change in waist‐to‐height ratio in 727 subjects.
**Figure S1.** Manhattan plot of GWAS on BMI *z*‐score.
**Figure S2.** Manhattan plot of GWAS on waist‐to‐height ratio.
