# Supplementary material for: Genetic risk score predicts risk for overweight and obesity in Finnish preadolescents
Source: Clin Obes. 2019 Oct 9;9(6):e12342. doi: 10.1111/cob.12342 (PMC6900004; doi:10.1111/cob.12342)

Supporting information

**Genetic risk score predicts risk for overweight and obesity in Finnish preadolescents**

Authors

Heli Viljakainen^1,2^, Emma Dahlström^1,3,4^, Rejane Figureido^1,5^, Niina Sandholm^1,3,4^, Trine B Rounge ^1,6^, Elisabete Weiderpass ^1,6-8*^

1 Folkhälsan Institute of Genetics, Folkhälsan Research Center, Helsinki, Finland

2 Department of Food and Nutrition, University of Helsinki, Helsinki, Finland

3 Abdominal Center, Nephrology, University of Helsinki and Helsinki University Hospital, Helsinki, Finland

4 Research Program for Clinical and Molecular Metabolism, Faculty of Medicine, University of Helsinki, Helsinki, Finland

5 Faculty of Medicine, University of Helsinki, Helsinki, Finland

6 Department of Research, Cancer Registry of Norway, Institute of Population-based Cancer Research, Oslo, Norway

7 Department of Medical Epidemiology and Biostatistics, Karolinska Institutet, Stockholm, Sweden

8 Department of Community Medicine, Faculty of Health Sciences, University of Tromsø, The Arctic University of Norway, Tromsø, Norway

* Currently affiliated at World Health Organization, International Agency for Research on Cancer, Lyon, France

Where authors are identified as personnel of the International Agency for Research on Cancer / World Health Organization, the authors alone are responsible for the views expressed in this article and they do not necessarily represent the decisions, policy or views of the International Agency for Research on Cancer / World Health Organization.

Corresponding author: Dr Heli Viljakainen, Folkhälsan Research Center, Topeliuksenkatu 20, 00250 Helsinki, Finland; Phone: +358 50 4485660; E-mail address: heli.viljakainen@helsinki.fi

Running title: Comparison of GRS with lifestyle factors

Contents

[Table S1: SNPs included in the BMI-GRS used in the study. 2](#_Toc17361190)

[Table S2: SNPs included in the WHR-GRS used in the study. 3](#_Toc17361191)

[Table S3: BMI-GRS associations with BMI z-score. 4](#_Toc17361192)

[Table S4: Background characteristics and change in anthropometric measures in 727 subjects by groups of under-/normal-weight (UN/NW) and overweight/obese (OW/OB) with mean (SD), if not indicated otherwise. 5](#_Toc17361193)

[Table S5: Associations of GRSs with change in BMI z-score and change in waist-to-height ratio in 727 subjects. 7](#_Toc17361194)

[Figure S1: Manhattan plot of GWAS on BMI z-score 8](#_Toc17361195)

[Figure S2: Manhattan plot of GWAS on waist-to-height -ratio 9](#_Toc17361196)

## Table S1: SNPs included in the BMI-GRS used in the study.

|  |  |  | Speliotes et al. 2010 | | | Finn-HIT cohort | | | | | |
| --- | --- | --- | --- | --- | --- | --- | --- | --- | --- | --- | --- |
| Chr. | **SNP** | **Gene** | **EA** | **EAF** | **Effect size** | **SNP** | **P_HWE_** | **EA** | **EAF** | **Effect size** | **P** |
| 1 | rs11165643 | *PTBP2* | A | 0.59 | 0.06 | rs1555543 (r^2^=0.98) | 0.90 | A | 0.61 | -0.02 | 0.68 |
| 1 | rs1514175 | *TNNI3K* | A | 0.43 | 0.07 | same | 0.63 | A | 0.47 | 0.11 | 0.006 |
| 1 | rs2815752 | *NEGR1* | A | 0.61 | 0.13 | same | 0.33 | A | 0.65 | 0.054 | 0.22 |
| 1 | rs543874 | *SEC16B* | G | 0.19 | 0.22 | same | 0.06 | G | 0.17 | 0.12 | 0.02 |
| 2 | rs11676272 | *RBJ* | G | 0.47 | 0.14 | rs713586 (r^2^=1.00) | 0.95 | G | 0.43 | 0.08 | 0.07 |
| 2 | rs2121279 | *LRP1B* | A | 0.18 | 0.09 | rs2890652 (r^2^=0.97) | 0.66 | A | 0.21 | -0.005 | 0.92 |
| 2 | rs2867125 | *TMEM18* | G | 0.83 | 0.31 | same | 0.34 | G | 0.83 | 0.17 | 0.003 |
| 2 | rs887912 | *FANCL* | A | 0.29 | 0.10 | same | 0.33 | A | 0.24 | 0.10 | 0.03 |
| 3 | rs13078807 | *CADM2* | G | 0.2 | 0.10 | same | 0.61 | G | 0.18 | 0.05 | 0.34 |
| 3 | rs7647305 | *ETV5* | G | 0.82 | 0.14 | rs9816226 (r^2^=0.84) | 0.07 | G | 0.82 | -0.03 | 0.63 |
| 4 | rs10938397 | *GNPDA2* | G | 0.43 | 0.18 | same | 0.55 | G | 0.47 | 0.09 | 0.04 |
| 4 | rs13107325 | *SLC39A8* | A | 0.07 | 0.19 | same | 0.26 | A | 0.02 | 0.18 | 0.25 |
| 5 | rs2112347 | *FLJ35779* | A | 0.63 | 0.10 | same | 0.72 | A | 0.58 | 0.03 | 0.41 |
| 5 | rs4836133 | *ZNF608* | A | 0.48 | 0.07 | NA | NA | NA | NA | NA | NA |
| 6 | rs206936 | *NUDT3* | G | 0.21 | 0.06 | same | 0.80 | G | 0.23 | 0.02 | 0.65 |
| 6 | rs987237 | *TFAP2B* | G | 0.18 | 0.13 | same | 0.26 | G | 0.22 | 0.02 | 0.71 |
| 9 | rs10968576 | *LRRN6C* | G | 0.31 | 0.11 | same | 0.95 | G | 0.38 | -0.01 | 0.79 |
| 11 | rs2030323 | *BDNF* | C | 0.78 | 0.19 | rs10767664 (r^2^=1.00) | 0.19 | C | 0.82 | -0.003 | 0.96 |
| 11 | rs3817334 | *MTCH2* | A | 0.41 | 0.06 | same | 0.85 | A | 0.40 | 0.04 | 0.39 |
| 11 | rs7127684 | *RPL27A* | G | 0.52 | 0.06 | rs4929949 (r^2^=0.98) | 0.15 | G | 0.53 | 0.07 | 0.09 |
| 12 | rs7138803 | *FAIM2* | A | 0.38 | 0.12 | same | 0.90 | A | 0.37 | 0.09 | 0.03 |
| 13 | rs4771122 | *MTIF3* | G | 0.24 | 0.09 | NA | NA | NA | NA | NA | NA |
| 14 | rs10134820 | *PRKD1* | A | 0.04 | 0.17 | rs11847697 (r^2^=1.00) | 1.00 | A | 0.02 | 0.14 | 0.38 |
| 14 | rs17109256 | *NRXN3* | A | 0.21 | 0.13 | rs10150332 (r^2^=1.00) | 0.52 | A | 0.24 | 0.01 | 0.88 |
| 15 | rs2241423 | *MAP2K5* | G | 0.78 | 0.13 | same | 0.40 | G | 0.83 | 0.11 | 0.06 |
| 16 | rs12444979 | *GPRC5B* | G | 0.87 | 0.17 | same | 0.48 | G | 0.88 | 0.02 | 0.74 |
| 16 | rs1421085 | *FTO* | G | 0.42 | 0.39 | rs1558902 (r^2^=1.00) | 0.23 | G | 0.43 | 0.11 | 0.01 |
| 16 | rs7359397 | *SH2B1* | A | 0.4 | 0.15 | same | 0.01 | A | 0.42 | 0.06 | 0.16 |
| 18 | rs571312 | *MC4R* | A | 0.24 | 0.23 | same | 0.63 | A | 0.19 | 0.10 | 0.06 |
| 19 | rs2287019 | *QPCTL* | G | 0.8 | 0.15 | same | 0.17 | G | 0.78 | 0.06 | 0.25 |
| 19 | rs29941 | *KCTD15* | G | 0.67 | 0.06 | same | 0.21 | G | 0.62 | -0.02 | 0.59 |
| 19 | rs3810291 | *TMEM160* | A | 0.67 | 0.09 | same | 0.30 | A | 0.65 | 0.06 | 0.16 |

## Table S2: SNPs included in the WHR-GRS used in the study.

|  |  |  | Heid et al. 2011 | | | Finn-HIT cohort | | | | | |
| --- | --- | --- | --- | --- | --- | --- | --- | --- | --- | --- | --- |
| Chr | **SNP** | **Gene** | **EA** | **Effect size** | **EAF** | **SNP** | **P_HWE_** | **EA** | **EAF** | **Effect**  **size** | **P** |
| 1 | rs10919388 | *GORAB* | C | 0.024 | 0.72 | same | 1 | C | 0.72 | 0.03 | 0.46 |
| 1 | rs905938 | *DCST2* | T | 0.025 | 0.74 | NA | NA | NA | NA | NA | NA |
| 2 | rs1385167 | *MEIS1* | G | 0.029 | 0.15 | same | 0.93 | G | 0.19 | -0.02 | 0.68 |
| 2 | rs1569135 | *CALCRL* | A | 0.021 | 0.53 | same | 0.40 | A | 0.56 | 0.02 | 0.72 |
| 3 | rs10804591 | *PLXND1* | A | 0.025 | 0.79 | same | 0.28 | A | 0.79 | -0.01 | 0.85 |
| 3 | rs17451107 | *LEKR1* | T | 0.026 | 0.61 | same | 0.55 | A | 0.67 | -0.03 | 0.48 |
| 4 | rs303084 | *SPATA5* | A | 0.023 | 0.80 | same | 0.38 | A | 0.84 | -0.02 | 0.78 |
| 4 | rs3805389 | *NMU* | A | 0.012 | 0.28 | same | 0.64 | A | 0.33 | -0.003 | 0.94 |
| 4 | rs9991328 | *FAM13A* | T | 0.019 | 0.49 | same | 0.08 | A | 0.51 | 0.11 | 0.01 |
| 5 | rs6556301 | *FGFR4* | T | 0.022 | 0.36 | same | 0.45 | A | 0.37 | 0.0005 | 0.99 |
| 5 | rs9687846 | *MAP3K1* | A | 0.024 | 0.19 | same | 0.61 | A | 0.77 | 0.03 | 0.54 |
| 6 | rs1776897 | *HMGA1* | G | 0.030 | 0.08 | same | 0.52 | C | 0.05 | 0.07 | 0.47 |
| 6 | rs7759742 | *BTNL2* | A | 0.023 | 0.51 | same | 0.03 | A | 0.47 | -0.04 | 0.39 |
| 7 | rs7801581 | *HOXA11* | T | 0.027 | 0.24 | same | 0.57 | A | 0.24 | -0.06 | 0.22 |
| 8 | rs12679556 | *MSC* | G | 0.027 | 0.25 | same | 0.19 | C | 0.23 | 0.04 | 0.41 |
| 8 | rs7830933 | *NKX2-6* | A | 0.022 | 0.77 | same | 0.59 | A | 0.79 | -0.05 | 0.32 |
| 9 | rs10991437 | *ABCA1* | A | 0.031 | 0.11 | same | 1 | A | 0.08 | -0.03 | 0.70 |
| 10 | rs7917772 | *SFXN2* | A | 0.014 | 0.62 | same | 0.90 | A | 0.59 | -0.05 | 0.21 |
| 11 | rs11231693 | *MACROD1* | A | 0.041 | 0.06 | same | 0.003 | A | 0.07 | -0.03 | 0.76 |
| 12 | rs4765219 | *CCDC92* | C | 0.028 | 0.67 | same | 0.30 | C | 0.69 | 0.07 | 0.14 |
| 15 | rs1440372 | *SMAD6* | C | 0.024 | 0.71 | same | 0.82 | G | 0.73 | -0.001 | 0.98 |
| 15 | rs8030605 | *RFX7* | A | 0.030 | 0.14 | same | 0.56 | A | 0.08 | 0.07 | 0.39 |
| 15 | rs8042543 | *KLF13* | C | 0.026 | 0.78 | same | 1 | G | 0.74 | -0.04 | 0.44 |
| 16 | rs2925979 | *CMIP* | T | 0.018 | 0.31 | same | 1 | A | 0.32 | 0.05 | 0.26 |
| 17 | rs4646404 | *PEMT* | G | 0.027 | 0.67 | same | 0.74 | G | 0.67 | 0.02 | 0.58 |
| 18 | rs8066985 | *KCNJ2* | A | 0.018 | 0.50 | NA | NA | NA | NA | NA | NA |
| 19 | rs12454712 | *BCL2* | T | 0.016 | 0.61 | same | 0.16 | A | 0.51 | 0.05 | 0.21 |
| 19 | rs12608504 | *JUND* | A | 0.022 | 0.36 | same | 0.20 | A | 0.33 | -0.06 | 0.23 |
| 19 | rs4081724 | *CEBPA* | G | 0.035 | 0.85 | same | 1 | G | 0.93 | -0.01 | 0.90 |
| 20 | rs224333 | *GDF5* | G | 0.020 | 0.62 | same | 0.76 | G | 0.59 | 0.03 | 0.44 |
| 20 | rs6090583 | *EYA2* | A | 0.022 | 0.48 | same | 0.14 | A | 0.51 | 0.06 | 0.15 |
| 20 | rs979012 | *BMP2* | T | 0.027 | 0.34 | same | 1 | A | 0.31 | 0.009 | 0.84 |

## Table S3: BMI-GRS associations with BMI z-score.

| GRS | N SNPs | Effect | 95%CI | R^2^ | SE | P |
| --- | --- | --- | --- | --- | --- | --- |
| zGRS_BMI_Speliotes_2010 | 30/32 SNPs found^1^ | 0.19 | 0.13-0.24 | 0.036 | 0.03 | 6.2 x10^-11^ |
| zGRS_BMI_Felix _2015 | 23/43 SNPs found^2^ | 0.14 | 0.09-0.20 | 0.021 | 0.03 | 5.8 x10^-7^ |
| zGRS_BMI_Locke_2015 | 76/77 SNPs found^3^ | 0.16 | 0.11-0.22 | 0.027 | 0.03 | 2.2e x10^-8^ |
| zGRS_BMI_Yengo_2018 | 308/941 SNPs found^4^ | 0.16 | 0.10-0.22 | 0.026 | 0.03 | 2.7 x10^-8^ |

^1^21 SNPs directly found, proxies (with r^2^ >0.80) found for 9 SNPs.

^2^20 SNPs directly found, proxies (with r^2^ >0.80) found for 3 SNPs.

^3^72 SNPs directly found, proxies (with r^2^ >0.80) found for 4 SNPs.

^4^308 SNPs directly found, proxies (with r^2^ >0.80) found for additional 155 SNPs (not included in GRS).

## Table S4: Background characteristics and change in anthropometric measures in 727 subjects by groups of under-/normal-weight (UN/NW) and overweight/obese (OW/OB) with mean (SD), if not indicated otherwise.

|  | UN/NW (BMIz ≤ +1) | | OW/OB (BMIz > +1) | | P^a^ |
| --- | --- | --- | --- | --- | --- |
| n | 555 | | 172 | |  |
| *Baseline* | Mean | SD | Mean | SD |  |
| Age, y | 11.3 | (0.2) | 11.3 | (0.1) | 0.26 |
| Weight, kg | 37.3 | (5.1) | 49.9 | (7.1) | < 0.001 |
| Height, cm | 148.2 | (6.6) | 150.7 | (6.5) | < 0.001 |
| BMI, kg/m^2^ | 16.9 | (1.5) | 20.4 | (6.6) | < 0.001 |
| BMI z-score | -0.1 | (0.7) | 1.6 | (0.4) | < 0.001 |
| Waist, cm | 63.6 | (4.7) | 75.5 | (7.5) | < 0.001 |
| Waist-to-height ratio (WtH) | 0.43 | (0.03) | 0.50 | (0.05) | < 0.001 |
| Parental BMI, kg/m^2^ (n=949) | 24.4 | (4.3) | 26.4 | (4.5) | < 0.001 |
| Weighted GRS of WHR, Z-scored | -0.02 | (1.01) | 0.06 | (1.05) | 0.359 |
| Weighted GRS BMI, Z-scored | -0.06 | (0.98) | 0.19 | (1.04) | 0.021 |
| *Follow-up* |  |  |  |  |  |
| Age, y | 15.3 | (0.4) | 15.4 | (0.4) | 0.423 |
| Weight, kg | 58.2 | (8.5) | 70.2 | (12.6) | < 0.001 |
| Height, cm | 170.3 | (8.4) | 171.1 | (8.5) | 0.259 |
| BMI, kg/m^2^ | 20.0 | (2.1) | 23.9 | (3.6) | < 0.001 |
| BMI z-score | 0.1 | (0.8) | 1.2 | (0.8) | < 0.001 |
| Waist, cm | 72.2 | (5.8) | 80.9 | (10.6) | < 0.001 |
| Waist-to-height ratio (WtH) | 0.42 | (0.03) | 0.47 | (0.06) | < 0.001 |
| △ age, y | 4.1 | (0.5) | 4.1 | (0.5) | 0.692 |
| △ weight, kg | 20.9 | (6.6) | 20.4 | (9.7) | 0.481 |
| △ height, cm | 22.1 | (6.8) | 20.4 | (6.6) | 0.005 |
| △ waist, cm | 8.5 | (5.5) | 5.6 | (8.3) | < 0.001 |
| △ BMI z-score, kg/m^2^ | 0.21 | (0.63) | -0.33 | (0.63) | < 0.001 |
| △ waist-to-height | -0.01 | (0.03) | -0.03 | (0.05) | < 0.001 |
|  | n | % | n | % |  |
| Gender |  |  |  |  | 0.094^b^ |
| Girls | 289 | 52.1 % | 77 | 44.8 % |  |
| Boys | 266 | 47.9 % | 95 | 55.2 % |  |
| Parental language (n=713) |  |  |  |  | 0.494^b^ |
| Finnish | 504 | 91.2 % | 152 | 91.0 % |  |
| Swedish | 31 | 5.7 % | 13 | 7.8 % |  |
| Other | 11 | 2.0 % | 2 | 1.2 % |  |
| Eating habit (n=673) |  |  |  |  | 0.855^b^ |
| Unhealthy | 64 | 12.4 % | 19 | 12.0 % |  |
| Vegetable and fruit avoider | 225 | 43.7 % | 73 | 46.2 % |  |
| Healthy | 226 | 43.9 % | 66 | 41.8 % |  |
| Leisure time physical activity (n=720) |  |  |  |  | 0.327^b^ |
| < 7 h per week | 259 | 47.2 % | 88 | 51.5 % |  |
| 7 h per week or more | 290 | 52.8 % | 83 | 48.5 % |  |
| Sleep duration during week (n=702) |  |  |  |  | 0.314^b^ |
| less than recommended | 14 | 2.6 % | 7 | 4.1 % |  |
| recommended | 519 | 97.4 % | 162 | 95.9 % |  |
| more than recommended | 0 |  | 0 |  |  |

WHtR; waist-to-height ratio, WHR; waist-to-hip ratio, GRS; genetic risk score

^a^ T-test

^b^ Chi-Square

##

## Table S5: Associations of GRSs with change in BMI z-score and change in waist-to-height ratio in 727 subjects.

|  | △ BMI z-score | | | | | |
| --- | --- | --- | --- | --- | --- | --- |
|  | Model | b | 95% CI | P | Adj R^2^ | SEE |
| Weighted BMI-GRS, Z-score | 1 | -0.063 | -0.113; -0.014 | 0.012 | 0.018 | 0.667 |
|  | 2 | -0.020 | -0.065; 0.025 | 0.382 | 0.200 | 0.602 |
| Weighted WHR-GRS, Z-score | 1 | -0.027 | -0.076; 0.021 | 0.267 | 0.011 | 0.670 |
|  | 2 | -0.015 | -0.058, 0.029 | 0.508 | 0.200 | 0.603 |
|  | △ waist-to-height ratio | | | | | |
| Weighted BMI-GRS, Z-score | 1 | -0.003 | -0.006; 0 | 0.042 | 0.038 | 0.038 |
|  | 2 | -0.001 | -0.004, 0.001 | 0.388 | 0.206 | 0.034 |
| Weighted WHR-GRS, Z-score | 1 | -0.001 | -0.004; 0.001 | 0.365 | 0.022 | 0.038 |
|  | 2 | -0.001 | -0.003; 0.002 | 0.678 | 0.205 | 0.034 |

b; b coefficient, Adj R2; adjusted R2 for the model, SEE; standard error of the estimate

Model 1; linear regression model adjusted for gender, age, sleep duration during week, eating habit, LTPA, parental language, PC1 and PC2.

Model 2; linear regression model adjusted for gender, age, sleep duration during week, eating habit, LTPA, parental language, PC1, PC2 and baseline BMI z-score/ waits-to-hip ratio.

## Figure S1: Manhattan plot of GWAS on BMI z-score


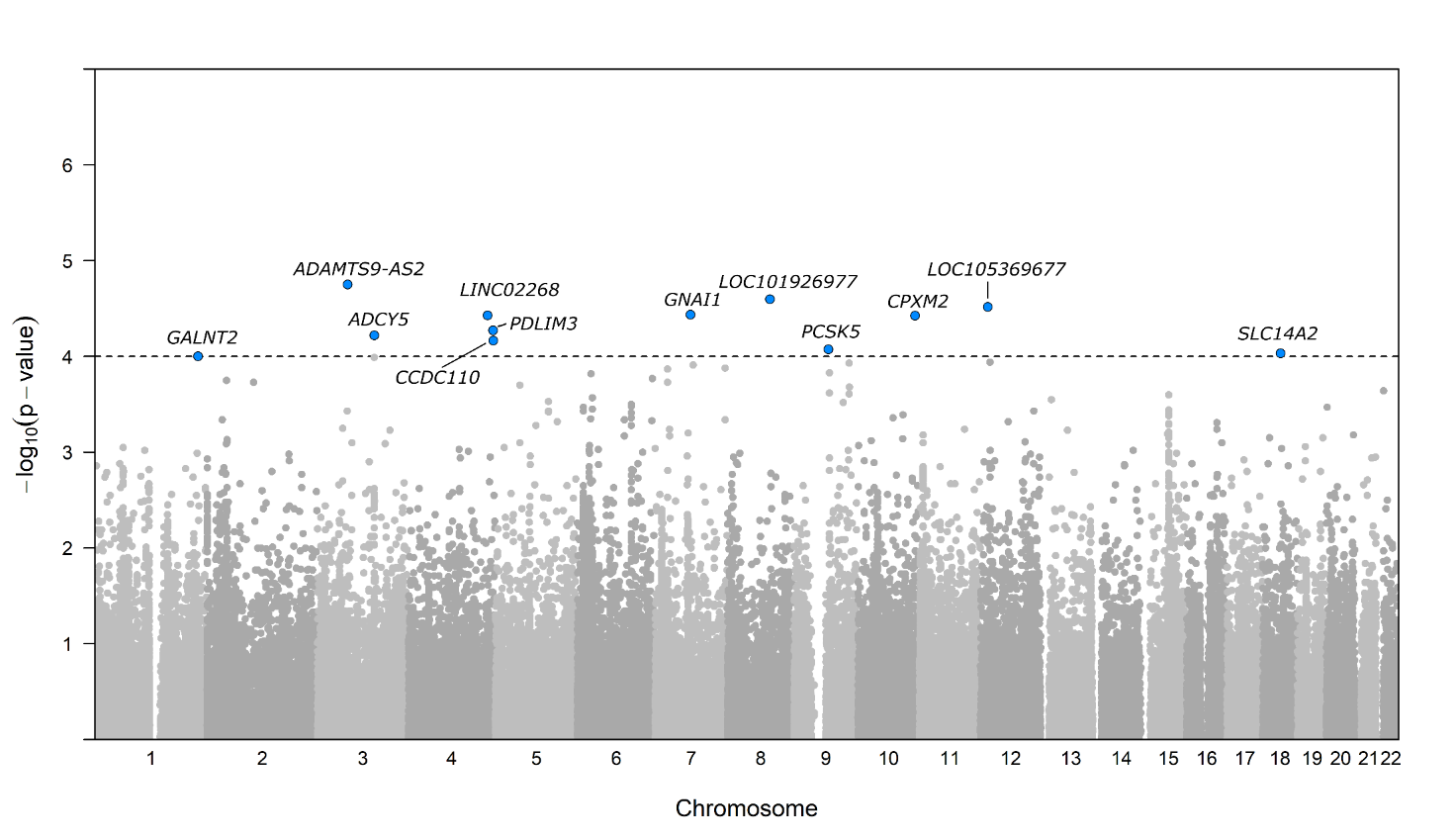


## Figure S2: Manhattan plot of GWAS on waist-to-height -ratio


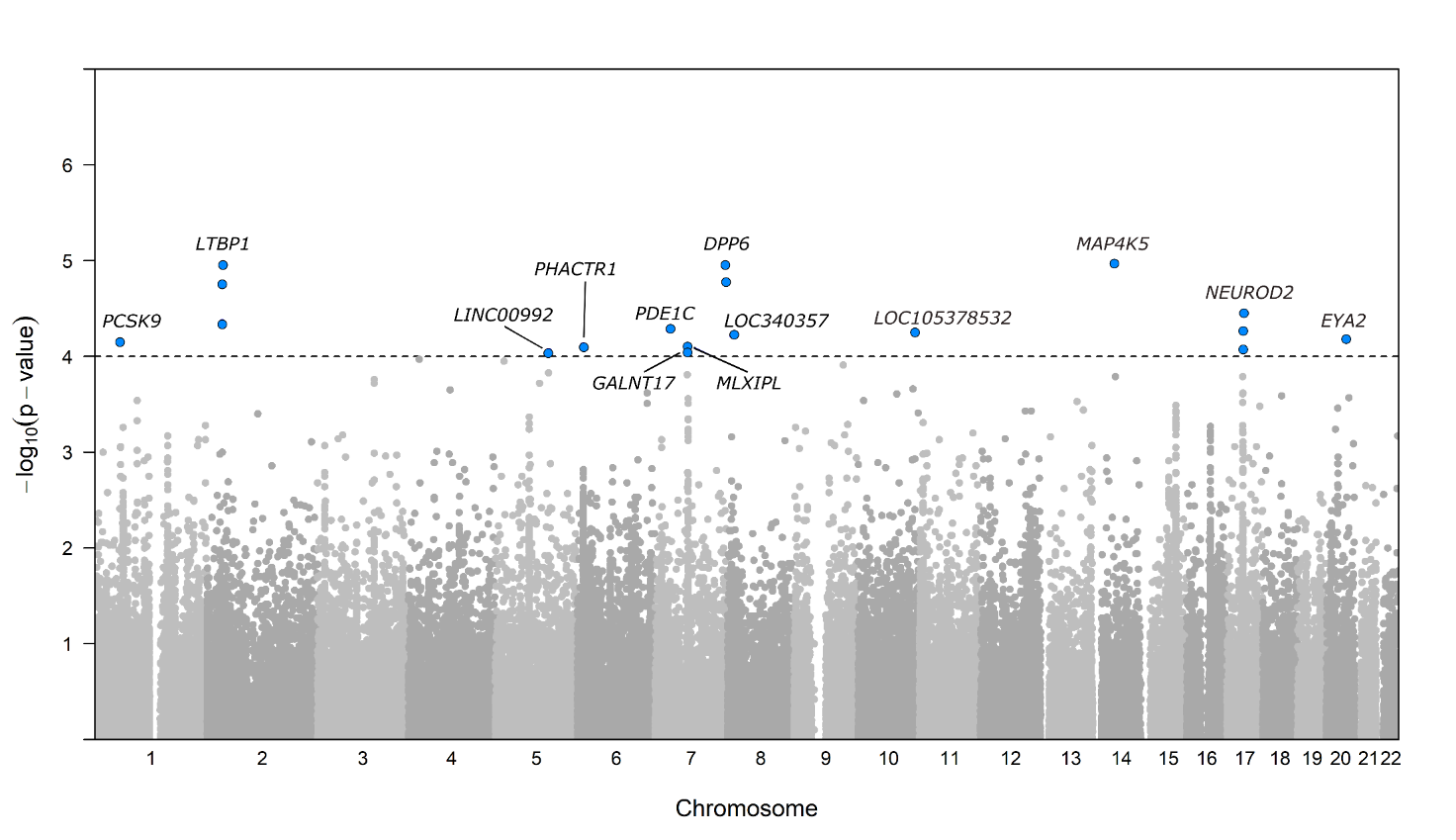

Supplement: Supplementary file 1 — Table S1. SNPs included in the BMI‐GRS used in the study. Table S2. SNPs included in the WHR‐GRS used in the study. Table S3. BMI‐GRS associations with BMI z‐score. Table S4. Background characteristics and change in anthropometric measures in 727 subjects by groups of under‐/normal‐weight (UN/NW) and overweight/obese (OW/OB) with mean (SD), if not indicated otherwise. Table S5. Associations of GRSs with change in BMI z‐score and change in waist‐to‐height ratio in 727 subjects. Figure S1. Manhattan plot of GWAS on BMI z‐score. Figure S2. Manhattan plot of GWAS on waist‐to‐height ratio. [file COB-9-na-s001.docx]
